# Structure-Functional Prediction and Analysis of Cancer Mutation Effects in Protein Kinases

**DOI:** 10.1155/2014/653487

**Published:** 2014-04-08

**Authors:** Anshuman Dixit, Gennady M. Verkhivker

**Affiliations:** ^1^Department of Pharmaceutical Chemistry, School of Pharmacy, The University of Kansas, 2010 Becker Drive, Lawrence, KS 66047, USA; ^2^Department of Biotechnology, Institute of Life Sciences, Bhubaneswar, India; ^3^School of Computational Sciences and Crean School of Health and Life Sciences, Schmid College of Science and Technology, Chapman University, One University Drive, Orange, CA 92866, USA; ^4^Department of Pharmacology, University of California San Diego, La Jolla, CA, USA

## Abstract

A central goal of cancer research is to discover and characterize the functional effects of mutated genes that contribute to tumorigenesis. In this study, we provide a detailed structural classification and analysis of functional dynamics for members of protein kinase families that are known to harbor cancer mutations. We also present a systematic computational analysis that combines sequence and structure-based prediction models to characterize the effect of cancer mutations in protein kinases. We focus on the differential effects of activating point mutations that increase protein kinase activity and kinase-inactivating mutations that decrease activity. Mapping of cancer mutations onto the conformational mobility profiles of known crystal structures demonstrated that activating mutations could reduce a steric barrier for the movement from the basal “low” activity state to the “active” state. According to our analysis, the mechanism of activating mutations reflects a combined effect of partial destabilization of the kinase in its inactive state and a concomitant stabilization of its active-like form, which is likely to drive tumorigenesis at some level. Ultimately, the analysis of the evolutionary and structural features of the major cancer-causing mutational hotspot in kinases can also aid in the correlation of kinase mutation effects with clinical outcomes.

## 1. Introduction

The human protein kinome presents one of the largest protein families that orchestrate functional processes in complex cellular networks during growth, development, and stress response [1–5]. The allosteric regulation of protein kinases serves as an efficient strategy for molecular communication and event coupling in signal transduction networks. Protein kinases are enzymes with a conserved catalytic domain that phosphorylates protein substrates and thereby play a critical role in cell signaling pathways [1–5]. Abnormal activation or regulation of protein kinases are major causes of human diseases, especially cancers. In fact, mutations in protein kinases often exemplify the phenomenon of “oncogene addiction,” whereby the structural effects of a specific set of mutations are necessary for a tumor to proliferate and hence have a selective advantage for tumor formation during somatic cell replication. As a result, protein kinases are important therapeutic targets for combating diseases caused by abnormal cell signaling [6–12]. Although the kinase catalytic domain is highly conserved, protein kinase crystal structures have revealed considerable structural differences between the closely related active and highly specific inactive forms of kinases [13–15]. The structures adopted by inactive kinases generally differ dramatically in the vicinity of the activation loop residues, in contrast to the well-conserved structures seen in active kinases [16–31]. Protein kinases interconvert between functionally important active and inactive states of the enzyme, and the phosphorylation of key residues can shift the balance between these states [13–15]. Evolutionary conservation and conformational plasticity of the kinase catalytic domain allow kinases to effectively achieve a dynamic equilibrium between active and inactive forms. This equilibrium ultimately facilitates regulation of their catalytic activity and recognition by other molecules. A steadily growing wealth of structural knowledge about the kinase catalytic domain and kinase complexes with inhibitors has demonstrated that protein kinase activity can be tightly regulated via dynamic interconversion between closely related active and highly specific inactive kinase states—a structural hallmark of the kinase domain which is critical for its normal function. What differentiates one kinase from another is the diversity of input signals that impinge on the catalytic domain, and a rich variation in the mechanisms that convert inactive forms of the kinase to active ones. The remarkable variability of kinase conformational states, which can include active, inactive, intermediate, and inactive-like conformations, has confirmed that diverse structures of the kinase activation loop may reflect natural kinase conformations and the dynamic equilibrium that occurs between them [27]. Thus, the interconversion between distinct inactive and active kinase states is an important characteristic feature of the kinase domain. Consequently, activating mutations that may perturb this equilibrium can result in an imbalance that can shift the kinase towards the active conformation and thus have a dramatic effect on the regulation of the enzyme. The Cancer Genome Atlas and related DNA sequencing initiative (http://www.cancergenome.nih.gov/) have motivated sequencing studies of tumors, all of which have produced initial results that suggest that the underlying genomic basis of tumorigenesis is complex [32–43].

Mutations in protein kinases, which are often implicated in many cancers, can exemplify the phenomenon of “oncogene addiction,” whereby the structural effects of a specific set of mutations are necessary for a tumor to proliferate and hence have a selective advantage for the formation of the tumor during somatic cell replication. A recent sequencing study of kinase coding regions in tumors attempted to differentiate which kinase gene mutations can cause the cancer phenotype (known as “driver mutations”) and which mutations are simply neutral mutational byproducts of somatic cell replication (known as “passenger mutations”) [38]. This study identified ~200 putative driver mutations among ~100 out of 254 kinases in 139 tumors. Another recent sequencing study described a systematic analysis of 13,023 well-annotated human protein-coding genes in 11 breast and 11 colorectal cancers in an initial “discovery” screen, followed by an analysis of 24 additional breast or colorectal tumors in a “validation” screen [39]. This study identified 189 genes displaying somatic mutations. The emerging mutational “landscape” of human cancers described in these papers suggests that only a few specific mutations are observed across different tumors (referred to as “mountains”) and many mutations appear to be common in small subsets of tumors [40]. The genomes of a malignant melanoma and a lymphoblastoid cell line have been sequenced from the same person, providing the first comprehensive catalogue of somatic mutations [41]. Other studies have classified tumor-associated somatic mutations according to their involvement in tumorigenesis by describing the mutational spectrum of 18, 191 distinct genes in 11 breast and 11 colorectal tumors through a systematic sequencing of exons.

Tumor sequencing efforts have identified a rich source of naturally occurring mutations with many being simple single nucleotide polymorphisms (SNPs) in kinases. A subset of these SNPs occurs in the coding regions (cSNPs) of kinases and results in a change in the encoded amino acid sequence (nonsynonymous coding SNP; nscSNPs). It is not clear, however, which of these nscSNPs actually contribute to tumorigenesis as “drivers.” Recent evidence suggests that cancer drivers have characteristics similar to Mendelian disease mutations [44]. Based on this information, a computational tool for predicting cancer-associated missense mutations, CanPredict, was developed [45]. Though quite powerful, generalized prediction methods such as CanPredict may fail to achieve the sensitivity and specificity attainable by prediction models tailored to individual protein families. We have also pursued a number of studies investigating the utility of sequence-based structural and evolutionary analysis of the effects of protein kinase nscSNPs using machine learning models [46–48]. A comprehensive computational analysis of the distribution of 1463 nscSNPs and 999 disease-causing nscSNPs within the kinase gene family has shown that disease-associated variations are overrepresented in the kinase catalytic domain [46]. A support-vector machine (SVM) analysis model was then developed to differentiate disease-associated nsSNPs from neutral nsSNPs in protein kinases [47]. By leveraging structural, phylogenetic, and physiochemical attributes, this method predicted known cancer driver mutations in protein kinases contributing to cancer progression. Using multiple subdomain based alignments, we have predicted that conserved positions harboring cancer-associated somatic mutations (CASMs) in multiple protein kinases contained a high proportion of predicted drivers, while kinase subdomains devoid of CASMs were more likely to contain passenger mutations [48]. In particular, we have identified a number of structurally conserved positions within the protein kinase catalytic core that appears to be frequent targets of tumorigenic mutations [48]. This suggests the existence of mutational hotspots, that is, conserved positions in the kinase catalytic domain, which may be statistically enriched in cancer and disease-causing mutations. We have also systematically catalogued disease and common SNPs, that is, those not known to cause disease, residing within the kinase catalytic core and then mapped them to individual subdomains, which are characterized by patterns of conserved residues [49]. It appears that the catalytic domain of protein kinases harbors a large number of disease causing nsSNPs as well as common or neutral nsSNPs that are not known to be associated with any diseases. We have found that while neutral kinase nsSNPs are randomly distributed within the catalytic core, known disease-causing nsSNPs are not directly involved in ATP binding but rather map to regulatory and substrate binding regions [49]. In contrast, cancer SNPs tend to lie closer to the catalytic machinery of protein kinases.

As an example of the importance of the characterizing the structural properties of genetic variations identified in tumors, consider that, for nonsmall cell lung carcinoma, systematic resequencing of tyrosine kinase genes has identified somatic mutations within the epidermal growth factor receptor (EGFR) tyrosine kinase gene [50–52]. The spectrum of lung cancer-derived EGFR mutations can induce oncogenic transformation by leading to constitutive kinase activity of EGFR and confer markedly different degrees of sensitivity to EGFR inhibitors. The discovery of activating mutations in the EGFR kinase domain and their differential sensitivity to inhibitors has suggested a potential structural divergence of the kinases in response to activating mutations and respective differences in the inhibitor binding [53–55]. Structural determination of the EGFR wild-type kinase and cancer mutants in complexes with inhibitors and interacting proteins has recently produced molecular insights into the functional effects of protein flexibility and relevant genetic modifications of this flexibility [56–59]. Using mutational analysis and crystallography it was discovered that the autoinhibited conformation of the EGFR kinase domain can resemble that of Src kinase and cyclin-dependent kinases [56, 57]. These structural studies have provided evidence that the active conformation previously observed in crystals of the EGFR kinase domain may have been a consequence of mimicry by the crystal (or by the inhibitor) of the intrinsic activation mechanism that can shift the dynamic equilibrium away from the otherwise stable inactive Src-like conformation. The structures of the wild-type EGFR kinase and mutants in complexes with a range of inhibitors have provided invaluable insights into the role of activating mutations in the EGFR kinase domain and their differential sensitivity to inhibitors [59]. The identification of EGFR mutations in a subset of human lung carcinomas and the association between EGFR mutation and drug sensitivity have suggested that genetic alterations in specific kinases and corresponding changes in structural and interaction profiles of kinases render tumors sensitive to selective inhibitors.

Some somatic mutations in kinases driving cancer may inactivate the catalytic activity of a kinase. In kinome-wide unbiased screens for kinase mutations in cancer, a large number of such inactivating mutations have been uncovered [60, 61]. The kinase-dead mutants of the catalytically important Asp residue from the DFG motif have been found in various kinases including BRAF [62, 63] as well as DAPK3, HCK, and LYN [60, 61]. The increasingly growing fraction of cancer driver mutations emerging from sequencing studies of protein kinase genes appeared to be inactivating or kinase dead leading to the loss of function. These studies have revealed that constitutively activating and kinase-dead mutations could play context-dependent opposing roles in cancer and may be simultaneously present in a variety of oncogenic kinases such as FGFR2/FGFR3 [64–68], MAP2 K4 [69], EPHA3 [70], DAPK3 [71], TRKB [72], ITK [73], and LKB1 [74]. These kinase-inactivating mutations have been found in various cancers and may represent a future class of drug targets.

Structural and computational approaches have been instrumental in revealing the atomic details of the protein kinase dynamics at different levels of complexity: from the detailed analyses of the catalytic domain to simulations of the regulatory dimer assemblies [56, 75–77]. In a pioneering computational study Shaw and colleagues have pushed the boundaries of simulation time scales to a new level revealing that oncogenic mutations in the EGFR kinase may reduce the disorder in the *α*C-helix region and in the dimerization interface, thereby facilitating the formation of asymmetric active EGFR dimer [75]. We have recently reported a series of computational investigations of oncogenic protein kinases that analyzed mechanisms of allosteric regulation in ABL and EGFR kinases by integrating multiscale simulations and modeling of long-range communications in the regulatory complexes [78, 79]. The results have unveiled organizing principles of mutation-induced activation in the ABL and EGFR kinases that may be orchestrated by a cross-talk between the integrating *α*F-helix and the mediating *α*C-helix, responsible for coordination of the interdomain coupling between key regulatory regions.

Despite significant advances in understanding structure and function of oncogenic mutations in protein kinases, a systematic analysis of structural and evolutionary characteristics of kinase-activating and kinase-inactivating cancer mutations across a significant fraction of human kinome is still lacking. In the current study, we employ a computational approach that combines sequence and structure-based prediction models to classify and characterize the effect of cancer mutations in protein kinases. We provide a detailed structural classification and analysis of functional dynamics for members of protein kinase families that are known to harbor cancer mutations. In particular, we characterize global features of functional dynamics for members of the EGFR, CDK, NEK, BTK, and ITK kinases that have various cancer mutations. Sequence and structure-based prediction models are then combined with the analysis of functional dynamics and collective motions to classify and characterize the effects of cancer mutations in protein kinases. Based on our analyses, we identify and characterize evolutionary conserved cancer-causing kinase mutational hotspots. We also focus on differential effects of activating and inactivating point mutations that allow modulating protein kinase activity. We show that kinase-inactivating mutations are often located in structurally rigid sites where mutations could either stabilize the autoinhibitory inactive state and prevent activation or severely reduce kinase activity through detrimental changes in the kinase fold. Evolutionary and structurally derived models of major cancer mutation hotspots are used to develop mechanistic understanding of mutation-induced regulation of kinase activity. The results of this study suggest that an understanding of the evolutionary and structural signatures of cancer-causing kinase mutations may provide a roadmap for the design of drugs that target specific mutational hotspots and thus facilitate an era of the precision medicine.

## 2. Results

### 2.1. Structural Analysis and Classification of the Protein Kinases with Cancer Mutants

The mechanisms that regulate catalytic activities of protein kinases can be categorized into phosphorylation, autoinhibition, and allosteric activation by binding partners [80–83]. Protein kinases are commonly regulated by phosphorylation of their activation loops that initiates a cascade of conformational changes enabling the kinase domain to adopt a catalytically competent conformation [82, 83]. For a number of kinases, however, phosphorylation may not be sufficient to guarantee the acquisition of biological activity. Some kinases may use more than one of these mechanisms to trigger functional transitions between inactive and active states. Mechanisms that prevent the untimely activation of kinases are crucial to ensure the proper cell division. Autoinhibition is one of these mechanisms in which kinases can adopt in the apo form an autoinhibited ordered conformation that prevents inappropriate kinase activity. Many protein kinases are regulated by structural mechanisms of autoinhibition, including EGFR, BRAF, CDKs, families of BTK, and NEK kinases. Structural coupling of the catalytic DFG motif and the regulatory *α*C-helix is recognized as central in controlling kinase activity and equilibrium between the inactive (DFG-out/*α*C-helix-in), the Cdk/Src-like inactive (DFG-in/*α*C-helix-out), and the active kinase forms (DFG-in/*α*C-helix-in) [83, 84]. The basic functional modes of autoinhibition are the binding of a regulatory domain to the catalytic domain and a conformation of the activation segment that blocks the nucleotide binding site. The defining feature of an autoinhibited conformation in protein kinases is that it can be reversed by activating mutation, leading to dramatic increases in kinase activity. Among protein kinases that fall into this category are most of the kinases harboring disease-causing mutations (both activating and inactivating).

**Figure 1:**
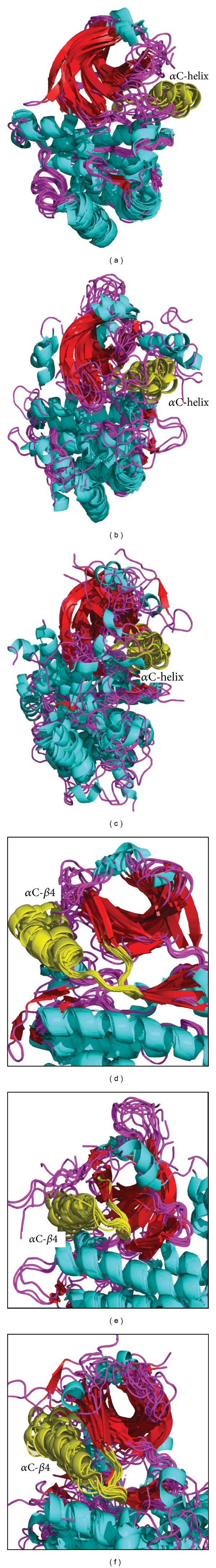
Structural analysis of the Cdk/Src kinases. The crystal structure alignment of the Cdk/Src kinases is presented separately for 3 groups of evolutionary related kinases. A diverse spectrum of the Cdk/Src kinases has a common structural determinant: the regulatory *α*C-helix assumes a *α*C-out conformation and prohibits the formation of the catalytically competent form. (a) The inactive crystal structures of Abl (pdb id 2G1T), Csk (pdb id 1BYG), Hck (pdb id 2HCK, 1AD5), Src (pdb id 2PTK, 1KSW, 1FMK), Btk (pdb id 3GEN, 3OCT), Itk (pdb id 3QGY, 1SM2), and BMX kinases (pdb id 3SXS). A close-up view of the *α*C-helix region in these structures (d). (b) The inactive structures of EGFR (pdb 1XKK, 2GS7), Her2 (pdb id 3PP0, 3RCD), Her3 (pdb id 3LMG,3KEX), Her4 (pdb id 2R4B, 3BBT, 3BCE), Tyk2 (pdb id 3NYX), Tie2 (pdb 2OSC), Met (pdb 2G15), Ron (pdb 3PLS), and Mer kinases (pdb id 2P0C). A close-up view of the *α*C-helix region in these structures (e). The remaining Cdk/Src crystal structures, representing distinct branches of the kinome tree, were assembled into one group. (c) The inactive structures of Cdk4 (pdb id 3G33), Cdk7 (pdb id 1UA2), Nek2 (pdb id 2W5H), Nek7 (pdb id 2WQM), MPSK (pdb 3DBQ), IRE1 (pdb id 2RIO), KSR2 (pdb id 2Y4I), ILK (pdb id 3KMU), WNK1 (pdb id 3FPQ), RSK1 (pdb id 2Z7S), OSR1 (pdb id 2VWI), MST4 (pdb id 3GGF), and CAMK1D (pdb 2JC6). A close-up view of the *α*C-helix region in these structures (f). The protein kinase structures are colored according to their secondary structure. The inactive position of the *α*C-helix-out is highlighted (colored in green). A close-up view highlights structural rigidity of the *α*C-*β*4 loop and certains variability of the *α*C-helix within the catalytically inactive position.

A common regulatory theme for one class of protein kinases with cancer mutations is based on sharing an intrinsically stable Cdk/Src structure and exhibiting a low catalytic activity of their isolated catalytic domains (Figure 1). Allosteric regulation of the Cdk/Src kinases occurs through targeted engagement of external binding partners that can perturb and destabilize the Cdk/Src inactive conformation, thus promoting activation [85, 86]. The Cdk/Src inactive structures are broadly distributed on the human kinome tree, covering all kinome groups, most notably TK, TKL, STE, CAMK, and CMGC families (Figure 1). The dominant cluster of the Cdk/Src kinases is located in the TK and TKL kinome groups and corresponds to the protein kinases with the Cdk/Src inactive conformation. A significant portion of the Cdk/Src-like kinases belongs also to the CMGC and STE protein kinase groups. A diverse spectrum of the Cdk/Src-like inactive structures is unified by a common structural determinant whereby the regulatory *α*C-helix is displaced outwards the N-terminal lobe and assumes an *α*C-out (swung-out) conformation, thus disallowing the formation of the catalytic salt bridge and preventing the formation of the catalytically competent form (Figure 1). Despite subtle differences in regulatory mechanisms, a high degree of structural conservation could be seen in the Cdk/Src inactive states for Abl, Csk, Hck, Src, Btk, Itk, and BMX kinases (Figures 1(a) and 1(d)). A greater conformational diversity was observed in another subgroup of the Cdk/Src structures, represented by members of the EGFR/ErbB tyrosine kinase subfamily (EGFR/Her1, ErbB2/Her2, ErbB3/Her3, and ErbB4/Her4 kinases), members of the HGFR tyrosine kinase subfamily (Met, Ron), and a member of the AXL tyrosine kinase subfamily (Mer) (Figures 1(b) and 1(e)). The remaining Cdk/Src crystal structures, representing distinct branches of the kinome tree, were assembled into one subgroup (Figures 1(c) and 1(f)). Structurally conserved features of the Cdk/Src kinases that determine their regulatory mechanisms are exemplified by structural rigidity of the *α*C-*β*4 loop that is linked to the positional variability of the *α*C-helix within the catalytically inactive state. The autoinhibitory interactions in the Cdk/Src structures typically involve the activation loop and the *α*C-helix that blocks the formation of the Lys-Glu catalytic salt bridge and prevent access to the active enzyme form. The critical features underlying kinase activation and stabilization of an active structural form include a dynamic assembly of the hydrophobic spine and consolidation of the Lys-Glu catalytic bridge [87, 88].

### 2.2. Functional Dynamics Maps of Protein Kinases Families with Cancer Mutations

**Figure 2:**
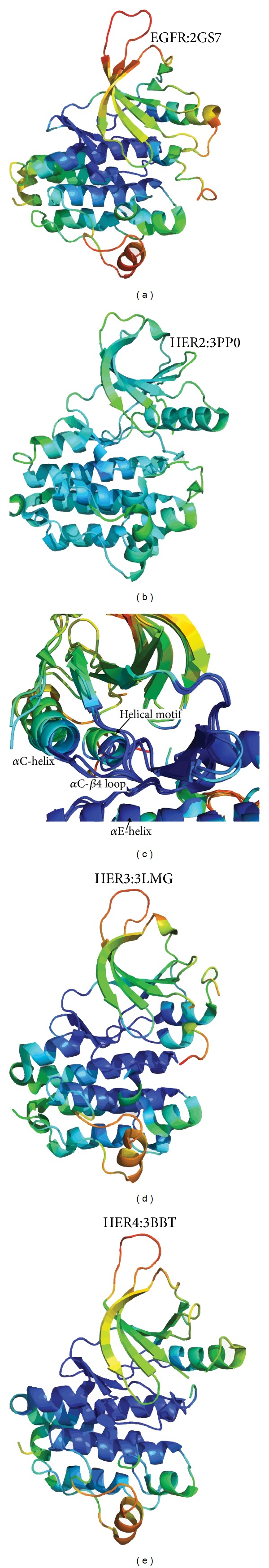
Functional dynamics and protein mobility maps of the EGFR subfamily of kinases. Conformational mobility maps of the Egfr/ErbB tyrosine kinase subfamily (EGFR/Her1, ErbB2/Her2, ErbB3/Her3, and ErbB4/Her4 kinases) were computed using MD simulations of the inactive crystal structures. Structural distribution of the protein kinase mobility is averaged over the two lowest frequency modes. A surface-based protein representation is employed, colored (blue-to-red) according to the protein residue motilities (from more rigid-blue regions to more flexible-red regions). A close-up of the regulatory hinge site formed by the *α*E-helix, *α*C-*β*4 loop, and the *α*C-helix is shown on the right panel. Structurally rigid *α*E-helix and *α*C-*β*4 loop are linked at the hinge site to a more flexible *α*C-helix. The autoinhibitory intramolecular clamp formed between the *α*C-helix and a short *α*-helix of the activation loop is a conserved feature of the EGFR kinases.

**Figure 3:**
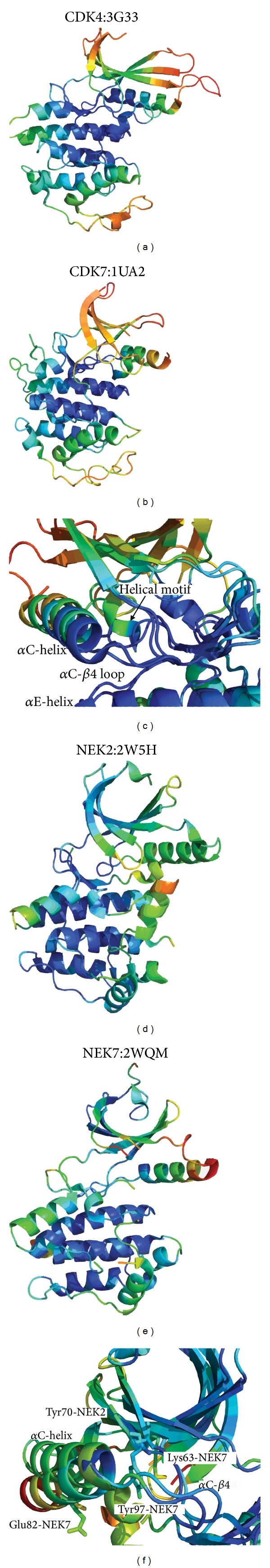
Functional dynamics and protein mobility maps of the Cdk and Nek subfamilies of kinases. Conformational mobility maps of the of the Cdk (Cdk4, Cdk7) (a, b, c) and Nek subfamilies (Nek2, Nek7) of kinases (d, e, f) were computed using MD simulations of the inactive crystal structures. Structural distribution of the protein kinase mobility is averaged over the two lowest frequency modes. A surface-based protein representation is employed and colored (blue-to-red) according to the protein residue motilities (from more rigid-blue regions to more flexible-red regions). A close-up of the regulatory hinge site formed by the *α*E-helix, *α*C-*β*4 loop, and the *α*C-helix is shown. The characteristic autoinhibitory interactions are formed between a small helical motif within the activation loop and the *α*C-helix that prevents access to the active enzyme form. The conserved functional residues (Tyr-70 in Nek2 and Tyr-97 in Nek7 shown in sticks on (f)) are projected from the *β*4 strand of the N-terminal right down into the hinge site between the *α*C-*β*4-loop and the *α*C-helix. These residues are implicated in regulatory interactions of Nek kinases with binding partners.

**Figure 4:**
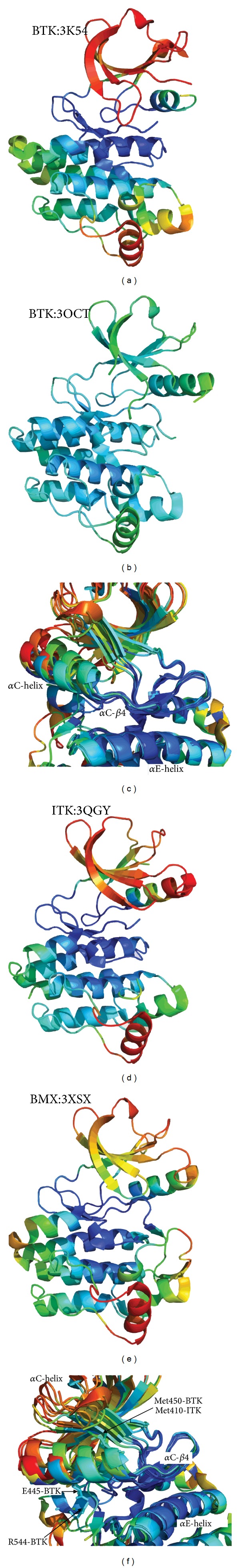
Functional dynamics and protein mobility maps of the Tec subfamily of kinases. Functional dynamics and conformational mobility maps of the Tec kinase subfamily (Btk, Itk, and Bmx kinases) were computed by using PCA of MD simulations of the inactive crystal structures. Structural distribution of the protein kinase mobility is averaged over the two lowest frequency modes obtained from all-atom MD simulations. A surface-based protein representation is employed and colored (blue-to-red) according to the protein residue motilities (from more rigid-blue regions to more flexible-red regions). Two different close-up views of the regulatory hinge site formed by the *α*E-helix, *α*C-*β*4 loop, and the *α*C-helix are shown on (a), (b), (c), (d), (e), and (f). The autoinhibitory electrostatic interactions between a helical motif within the activation loop (Btk-R544 is shown in sticks) and the *α*C-helix (Btk-E445 is shown in sticks) are shown on (f). The conserved Btk-M450 and Itk-M410 located near the regulatory hinge site (shown in sticks on the right lower panel) are critical activating residues.

In this section, we describe a comparative analysis of conformational dynamics performed for a significant number of protein kinases with known cancer mutations. We establish connections between global motions, regulatory activation mechanisms, and functional requirements to harbor cancer mutations. The principal collective motions of protein kinases and kinase mutant structures were analyzed using molecular dynamics (MD) simulations and principal component analysis (PCA) of MD trajectories. The structural distribution of the protein mobility and the cross-correlation maps of protein residue fluctuations were computed along the dominant principal component modes. This analysis identified the shapes of the low frequency modes and the protein regions subjected to correlated and/or anticorrelated motions along the selected low frequency modes. A typical conformational mobility map of a Cdk/Src-like kinase along low frequency modes is characterized by a network of connected structurally stable elements mainly determined by the interactions between the *α*E-helix of the C-terminal lobe and the *α*C-*β*4-loop motif of the N-terminal lobe (Figures 2–4). Structural analysis of the protein kinases with a Cdk/Src-like conformation shows a commonly displaced *α*C-helix that can maintain a certain positional variation within the “swung-out” form. A structurally rigid *α*C-*β*4-loop linked to a relatively mobile *α*C-helix presents a “dynamic signature” of the Cdk/Src-like kinases that is likely to play role in regulating functional transitions between inactive “*α*C-out” and active “*α*C-in” positions. The boundary between the *α*E-helix/*α*C-*β*4-loop region and the *α*C-helix can define a functional hinge connecting structurally rigid and flexible regions of the N-terminal lobe that would guide coordinated collective motions of the kinase catalytic domain. Structurally rigid and conformationally flexible residues may often occupy proximal positions to the hinge sites in allosteric protein systems, where the residues located at the “border” between regions of high and low structural stabilities could form recognition sites and control global protein movements. Based on this evidence, a functional region at the junction of the *α*C-helix and the adjacent *α*C-*β*4-loop may be responsible for principal motions of the Cdk/Src-like kinases during activation mechanisms.

### 2.3. Conformational Dynamics Profiles of EGFR Kinases

Functional motions of the Cdk/Src-like kinases have displayed commonalities and subtle differences in the dynamics of the *α*C-*β*4 and *α*C-helix motifs that are unified by a common functional goal destabilization of the Cdk/Src inactive state and stabilization of the active state through interactions with binding partners or activating mutations. Kinase activation through an asymmetric head-to-tail dimerization of catalytic domains is a common regulatory scenario of the EGFR/ErbB subfamily of tyrosine kinases [89–95]. The autoinhibitory intramolecular clamp formed between the *α*C-helix and a short *α*-helix of the activation loop is a conserved feature of the Cdk/Src inactive structure shared by all EGFR kinases (Figure 2). Functional dynamics of the inactive Her3 [96, 97] and Her4 kinases [98, 99] highlighted the intrinsic structural stability of the autoinhibitory enzyme form (Figure 2). Despite a shortened *α*C-helix in the crystal structures of the Her3 receptor [96, 97], the broadened “rigid-blue” area of the catalytic core around the *α*C-*β*4/*α*C-helix region could lock the receptor in a stable Cdk/Src-like inactive form (Figure 2). The obtained dynamic profile of the Her3 kinase corroborates with structural analyses by Kuriyan's [96] and Lennon's groups [97] who attributed the lack of Her3 catalytic activity to an overly stable inactive form. Somewhat of a “family outlier” is the Her2 kinase [100], a kinase with the increased conformational flexibility of the inactive structure that is spread beyond the *α*C-helix region.

### 2.4. Conformational Flexibility Profiles of CDK and NEK Kinases

The protein kinases harboring cancer mutations are often regulated by similar activation mechanisms and are involved in a similar cellular function. Mitotic phosphorylation events in the cell can be catalyzed by members of the Cdk [101, 102] and Nek families [103–105] that are activated by structurally similar mechanisms (Figure 3). The Cdk kinases (Cdk2, Cdk4, and Cdk7) live by default in their stable inactive Cdk/Src states and are activated through a cascade of signaling events that include binding to their respective cyclin partners: Cdk2-cyclin A [85, 86] and Cdk4-cyclin D1 [101, 102]. While cyclin binding is sufficient to trigger activation of Cdk2, the crystal structure of Cdk4 bound to cyclin D1 revealed an inactive kinase conformation (Figure 3) that remains thermodynamically stable even in the presence of the binding partner. Structural studies suggested that the Cdk4-cyclinD1 complex could present an intermediate state in which Cdk4 is potentiated for final activation [101]. Functional dynamics of Cdk4 and Cdk7 kinases showed subtle but important differences between these closely related kinases, particularly revealing a greater rigidity of the inactive Cdk4 state that is spread through the entire structural core and the *α*C-*β*4/*α*C-helix region (Figure 3).

Nek2, Nek7, and Nek9 are members of the human Nek family [103–105] that are involved in mitotic function. Activation of Nek kinases combines phosphorylation and dimerization-dependent allosteric regulation by engaging members of its own family as binding partners [106]. The crystal structures of human Nek2 [106, 107] and Nek7 [108] revealed a Cdk/Src-like inactive structure with characteristic autoinhibitory interactions between a small helical motif within the activation loop and the *α*C-helix that blocks the formation of the Lys-Glu catalytic bridge and prevents access to the active enzyme form (Figure 3). This autoinhibitory clamp represents a common structural solution to a stable inactive state that is also shared by Cdk and EGFR families of kinases. Structural studies demonstrated that Nek7 can be activated by engaging the noncatalytic domain of Nek9 in targeting and remodeling the vicinity of the *α*C-*β*4-loop [108]. Consistent with these experimental insights, we find that functional dynamics of the *α*C-*β*4 region and the activation loop are similar in Cdks and Neks, where a structurally rigid *α*C-*β*4-loop is coupled to dynamic motions of a more flexible *α*C-helix and the autoinhibitory helix of the activation loop (Figure 3). Interestingly, the intramolecular clamp in Cdk kinases is structurally more rigid as compared to Neks (refer to a larger “blue” area near the autoinhibitory helix in Figure 3). On other hand, the autoinhibitory small helix in Nek kinases is less stable (Figure 3) and together with the *α*C-helix forms a flexible component of the regulatory hinge that is prone to functional changes. Hence, regulatory interactions of Nek kinases with binding partners may be readily facilitated by the coupled dynamics of the *α*C-*β*4 and *α*C-helix motifs. This model is supported by structural studies of Neks that pointed to a critical activation-promoting role of the conserved tyrosine (Tyr-70 in Nek2 and Tyr-97 in Nek7) that is projected from the *β*4-strand of the N-terminal right down into the “border” area between the *α*C-*β*4-loop the *α*C-helix (Figure 3).

### 2.5. Conformational Mobility Maps of TEC Kinase Family

The Tec family is a subfamily of nonreceptor type tyrosine kinases and includes Btk, Itk, and Bmx kinases [109]. These kinases share a common SH3-SH2-kinase organization also found in the Src, Hck, Csk, and Abl kinases [110], yet they are regulated in an opposite manner. The disengagement of the SH2-SH3 domains relieves the autoinhibitory constraints in c-Src and Hck kinases and yields an activated form [110]. In contrast, the catalytic domains of Btk, Itk, and Bmx kinases adopt the Cdk/Src-like inactive structure and require binding to the SH2-SH3 assembly for activation. The crystal structures of the Btk, Itk, and Bmx catalytic domains in complexes with inhibitors indicated that these kinases can be stabilized in a number of distinct protein conformations that represent variations of the catalytically inactive Cdk/Src-like structure (Figure 4) [111–116]. The autoinhibitory electrostatic interactions between a helical motif within the activation loop (Btk-R544) and the *α*C-helix (Btk-E445) protect the stability of the inactive state (Figure 4). Functional dynamics of the Tec kinases in their Cdk/Src-like inactive state displayed a common protein mobility profile. It is worth noticing, however, a uniform structural rigidity of the *α*E-helix and the *α*C-*β*4 loop in all Tec kinases. This contrasted with a fairly mobile *α*C-helix as evident from our dynamic analysis and reflected in the positional variability seen in the crystal structures (Figure 4).

### 2.6. Structural and Functional Signatures of Cancer Mutations in Protein Kinases

**Figure 5:**
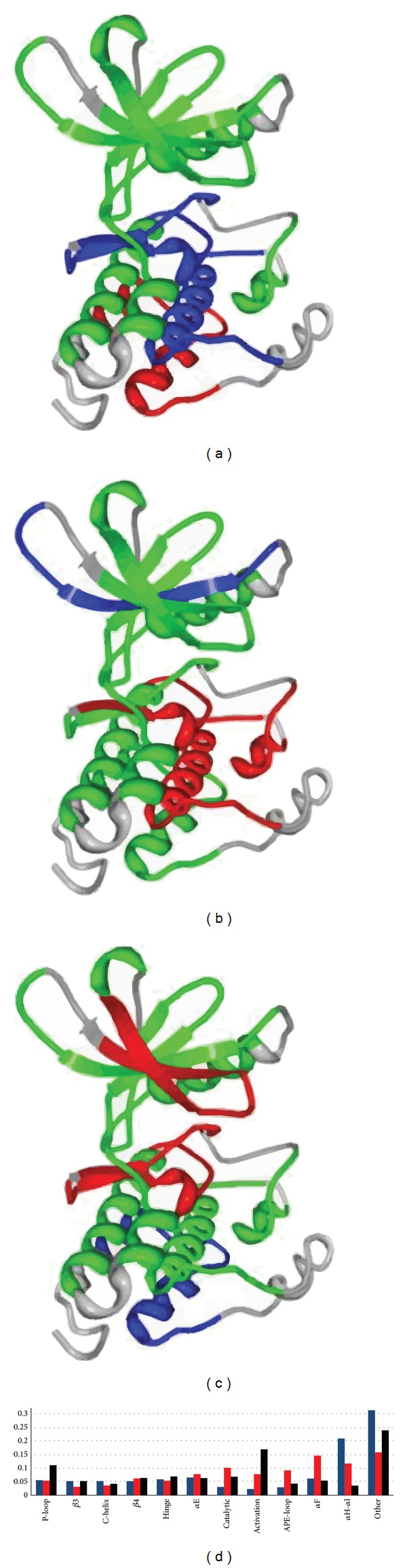
Structural distribution of protein kinase SNPs. The structural distribution of common SNPs (a), congenital disease-causing SNPs (b), and cancer-associated SNPs (c) is depicted along with the frequencies of SNPs within specific structural domains (d). In (a)–(c), green coloration represents regions with an SNP frequency equivalent to what would be expected by random chance, blue coloration represents regions statistically devoid of SNPS, and red coloration depicts regions statistically enriched in SNPs. Note that regions enriched and devoid of SNPs are different across the three mutation types, with minimal overlap. In (d) blue bars represent common SNPs, red bars represent disease causing SNPs, and black bars represent cancer associated SNPs.

The catalytic domain of protein kinases harbors a large number of SNPs falling into three major categories: common and likely neutral SNPs, inherited disease causing (i.e., germline) SNPs, and cancer causing (i.e., somatic) SNPs. By compiling and mapping a total of 355 common SNPs, 428 inherited disease causing SNPs, and 541 cancer associated SNPs to the catalytic core of protein kinases [117, 118], we find an enrichment of different categories of SNPs in the different structural regions of the catalytic domain (Figure 5). The widely varying distributions among the three mutation types suggest that the different SNP types result in different types of functional consequences. Common SNPs sparsely populate important functional segments such as the catalytic and activation loops, while densely populate the C-terminal tail. Importantly, common SNP hotspots occur at evolutionarily unconserved positions of the C-terminal region. In contrast, inherited disease-causing SNPs populate regions which are involved in regulation and substrate binding, such as the APE-loop and the P+1 binding pocket, as well as the catalytic loop (Figure 5). In turn, cancer associated SNPs strongly target catalytic and nucleotide binding functions localizing to the P-loop, as well as activation and catalytic segments of the catalytic core. Though the regions enriched in cancer SNPs contain highly conserved residues, cancer SNPs reveal a relatively modest conservation pattern relative to disease SNPs and a more diffuse distribution across the catalytic core (Figure 5). There are overlaps in structural distribution for the three SNP types, but the statistically enriched regions are nearly completely distinct, presumably confirming the differences in the functional effects of these three mutational types.

**Figure 6:**
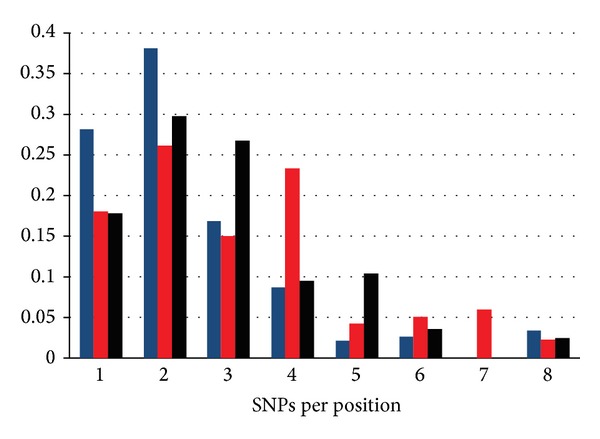
The position-specific distribution of SNPs. The number of common (blue), disease (red), and cancer (black) SNPs per position is plotted against the frequency of SNPs occurring at structurally identical positions. Note that the position-specific distribution of cancer SNPs appears to be a hybrid of common and disease SNPs.

The contribution of sequence conservation to the evolutionary signature of cancer mutations and the overall functional differences across all three SNP types is also mirrored in the number of SNPs occurring at structurally equivalent positions across the protein kinase family (Figure 6). Common SNPs, which are assumed to have little functional affect, are randomly distributed throughout the catalytic core. Therefore, the structural distribution of common SNPs is dominated by positions mutated in a single, or very few protein kinases. On the other hand, of the three types of SNPs, inherited disease-causing SNPs tend to be more highly concentrated at structurally equivalent positions, with a significant excess of mutations occurring at positions mutated in four or more different protein kinases (Figure 6). This can be explained by the preference of inherited disease SNPs for residues involved in substrate binding, allosteric, or regulatory functions, where kinase activity is retained, as is viability, but producing a biological deficit. Given that somatic SNPs occur randomly and uniquely in each given tumor, followed by selection for tumor cells with a growth advantage, the position-specific distribution of cancer SNPs is similar to common SNPs but is shifted towards a higher number of SNPs per position, probably due to the selection of tumorigenic mutational hotspots which are shared across multiple protein kinases.

**Figure 7:**
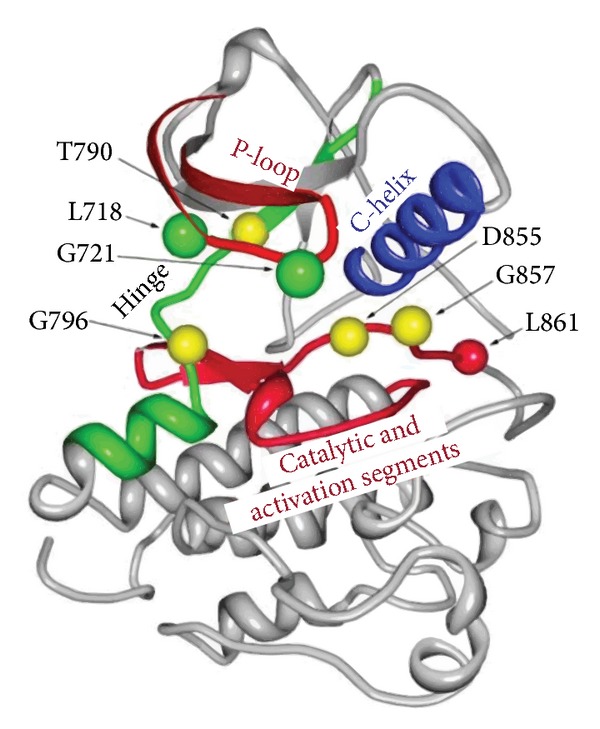
Structural mapping of cancer mutational hotspots in protein kinases. The mutational hotspots are displayed using the EGFR kinase (pdb entry 1M14). The C-helix devoid of cancer mutations and drivers is shown in blue. The green hinge region contains an excess of predicted driver mutations, and the red catalytic and activation loops contain an excess of both cancer mutations and predicted drivers. Green balls denote residues with 4 predicted driver mutations, yellow balls denote residues with 5 predicted driver mutations, and the red ball denotes L861, which is predicted to be a driver in eight different kinases.

Somatic mutations occurring at structurally conserved positions within the protein kinase domain tend to be statistically enriched in tumors and cluster into specific mutational hotspots. These cancer mutation hotspots occur in functionally important protein kinase segments (Figure 7), containing an abundance of predicted cancer driver mutations. Segments involved directly in catalytic functions, such as the P-loop, catalytic loop, and activation loop tend to be populated by cancer-causing mutations. On the other hand, the C-helix, which undergoes large movements when transitioning from the active to inactive conformation, is significantly devoid of cancer mutations. The hinge region is an intermediate case, where the segment does not appear to contain an excess of cancer mutations. Evolutionary analysis has identified that cancer mutation hotspots can cluster in structurally conserved positions corresponding to L858, L718, G721, T790, G796, D855, G857, and L861 in EGFR (Table 1), which are localized within the P-loop, hinge region, and activation loop (Figure 7).

We specifically focus on cancer mutation hotspots which have been identified as a frequent target of tumorigenic activating and inactivating mutations. A detailed structural and conformational dynamics analysis was conducted for major mutational hotspots of kinase-activating and kinase-dead mutations, namely, (a) conserved hotspots corresponding to activating cancer mutants L858R and L861Q in EGFR and (b) a conserved hotspot corresponding to the catalytic Asp residue from the DFG motif implicated in kinase-dead mutations in BRAF, DAPK3, HCK, and LYN (Table 1).

Based on these findings, we embarked on a detailed comparative analysis of activating and inactivating mutations on structure and dynamics of various kinase genes including EGFR, BRAF, FGFR2, FGFR3, MAP2K4, EPHA3, DAPK3, and TRKB kinases (Table 2) and we propose that structural and dynamic signatures of cancer mutations may provide a clue to the underlying mechanism of kinase activation. We test one of our hypotheses that the mechanism of activating mutations may result from a combined effect of the partial destabilization of the kinase in its inactive state and a concomitant stabilization of its active-like form. We also suggest that activating and inactivating mutations may differentially target the catalytic domain based on structural stability and flexibility of the kinase residues.

### 2.7. Structural Analysis and Conformational Dynamics of the EGFR Cancer Mutants

**Figure 8:**
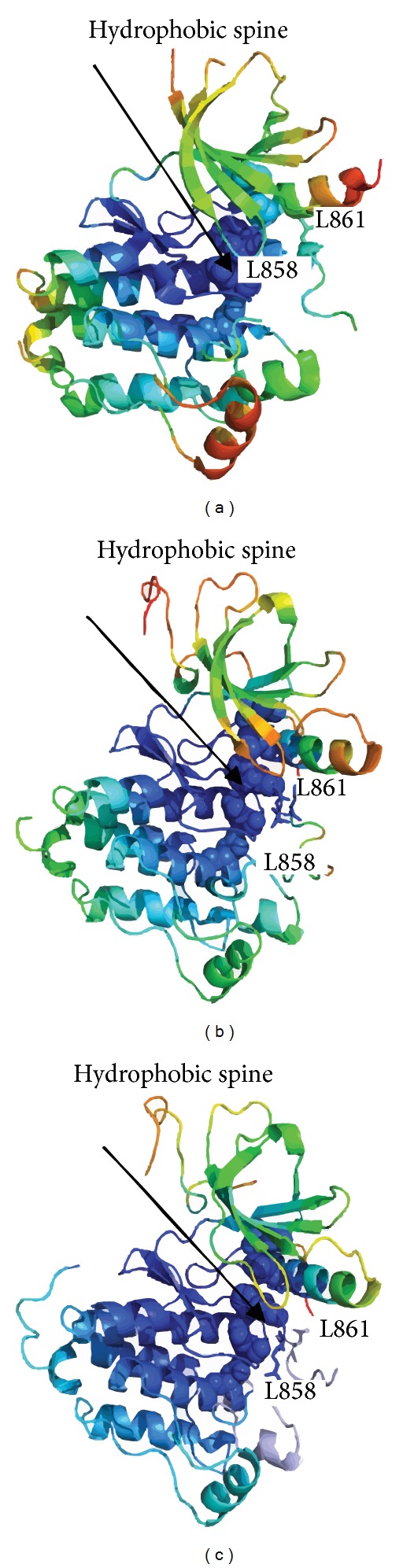
Structural modeling and dynamics profile of L858R EGFR mutant. (a) The dynamics profile of the inactive wild-type EGFR (pdb id 1XKK). (b) The dynamics profile of the active wild-type EGFR (pdb id 2J6M) crystal structure of the wild-type EGFR. (c) The structural model and dynamics profile of the L858R mutant. The L858, L858R, and L861 residues are shown in sticks and colored according to their mobility profile. The hydrophobic regulatory spine residues (M766-L777-H835-F856-D896) are shown in spheres. Structural distribution of the protein kinase mobility is averaged over the two lowest frequency modes obtained from all-atom MD simulations. A surface-based protein representation is employed, colored (blue-to-red) according to the protein residue motilities (from more rigid-blue regions to more flexible-red regions).

Here we report the results of structural modeling and dynamics analysis of the activating cancer mutations in EGFR L858R and L861Q (Tables 1 and 2). An activating mutation in the activation loop of the EGFR kinase domain, L858R (also identified as Leu834 in a different numbering of the EGFR sequence), is among most frequent mutations in lung cancer, amounting to more than 40% of EGFR mutations in this cancer category, and can result in a dramatic enhancement of EGFR activity [50–52]. The crystal structures of EGFR-L858R and EGFR-T790M [59] have shown that these cancer-causing modifications could stabilize the active kinase form. We pursued structural modeling of L858R mutant starting from both inactive (pdb entries 1XKK, 2GS7) and active wild-type EGFR structures (pdb entries 2J6M, 2ITX, 2ITW, and 2ITY). The predicted structural models and dynamics analysis of the L858R EGFR mutant conforms rather closely to the mutant X-ray structure (pdb entry 2ITT), moving considerably away from the initial wild type crystal structures and accurately reproducing the conformational rearrangement of the activation loop towards the active-like state (Figure 8) [59].

**Figure 9:**
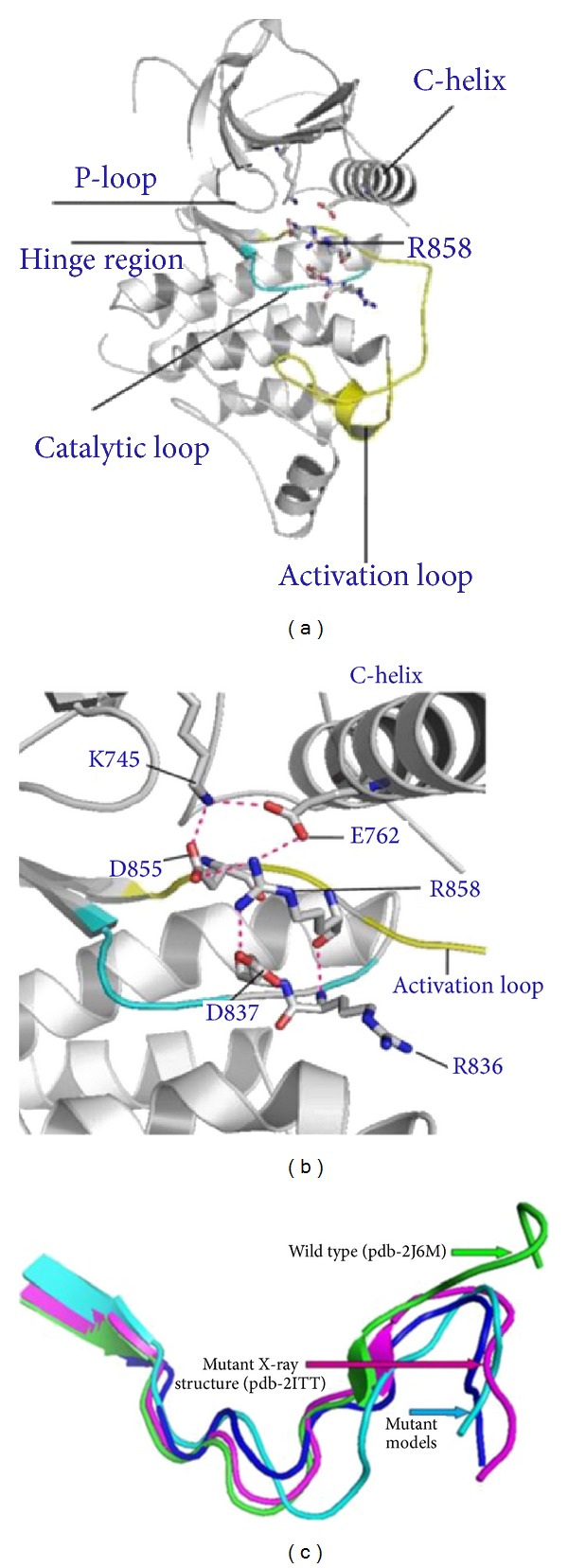
Computational modeling of the EGFR-L858R mutant. (a) The predicted structure of the L858R EGFR mutant. (b) A close-up of functionally important residues and interactions stabilizing the EGFR mutant. (c) A close-up comparison between conformations of the activation loop in the wild-type EGFR crystal structure (pdb entry 2J6M, green), L858R mutant crystal structure (pdb entry 2ITT, pink), and two best predicted mutant structures. The activation loop conformation of the two lowest energy predicted models of EGFR-L858R are shown in blue and cyan. The first predicted model of the complete EGFR-L858R protein structure has RMSD = 1.98 Å from the crystal structure (with the activation loop in blue). The second lowest energy model of the complete EGFR-L858R structure is within RMSD = 2.52 Å from the crystal structure (with the activation loop in cyan).

In agreement with structural experiments [27], we found that EGFR-L858R may be incompatible with the immediate hydrophobic environment in the inactive structure which disfavors a polar side chain of EGFR-L858R. The mobility profiling of the inactive wild-type EGFR (Figure 8(a)) showed that L858 and L861 are located in the regions that are only marginally stable and therefore the introduction of the energetically unfavorable side chains would destabilize the inactive form and facilitate a transition to the active kinase form. In the crystal structure of the wild-type EGFR in the active state, Leu858 and L861 are surface exposed, with the backbone carbonyl oxygen of Leu858 making hydrogen bond interactions with Arg836 and the ion pair between Lys745 and Glu762 intact in this conformation (Figure 8(b)). The effect of the L858R mutation results in destabilization of the inactive conformation and stabilization of the active state by enhancing rigidity of the *α*C-helix regulatory segment. An inspection of the predicted structural model indicated that important interactions formed in the crystal structure of EGFR-L858R could be adequately reproduced, including a stable K745-E762 ion pair, known as a critical attribute of the active kinase form. The key interactions which stabilize the L858R mutant in the active state are made by the side chain of Arg858, which becomes ordered and forms a hydrogen bond with the main chain carbonyl of Arg836 as well as the hydrogen bond between Lys745 and Glu762 (Figure 9). In the L858R mutant model, Arg858 forms an extensive hydrogen bond network which includes interactions with Asp837 (catalytic loop), Glu762 (*α*C-helix), and Asp855 (activation loop) (Figure 9). Hence, we observed that, in the EGFR-L858R mutant, the substitution of the hydrophobic Leu in the activation loop with a charged residue results in the stabilizing interactions with the residues from the *α*C helix (Figure 8(c)). The structural mobility map of the L858R mutant revealed the increased rigidity (marked by propagation of the blue color) that is spread beyond the mutational site and resulted in stabilization of the activation loop as well as the interactions between L858R and the *α*C-helix. It could be also seen that the *α*C-helix conformation becomes more stable in the active form (Figure 8(c)). The L858R mutation was originally proposed to function by destabilizing the inactive conformation of the activation loop, thus inducing thermodynamic stabilization of the constitutively active conformation [56, 59]. In the most recent long-time scale simulation, an indirect activation mechanism was suggested according to which L858R transformation could suppress the disorder in the *α*C-helix region, thus favoring dimerization and formation of the active dimer [75]. Here, we presented the large scale dynamics analysis of the kinase catalytic domain for many kinases and cancer mutants. Although our analysis of EGFR-L858R is based on simulations of the catalytic domain, we found that stabilizing effects favoring the formation of the active catalytic domain conformation and asymmetric dimerization stabilizing the interfacial *α*C-helix region are synergistic and cooperative in nature reactions that act collectively to produce the phenotypic response. In other words, the observed formation of the active kinase conformation, accompanied by partial reduction of flexibility in the *α*C-helix, presents a necessary prerequisite for asymmetric dimerization and formation of the active dimer seen in [75]. To summarize, our results are consistent with these studies and offer additional important insights concerning synergistic nature of different stages involved in the activation mechanism. The global effect of these steps is to shift the balance toward the active state and favor the formation of the asymmetric dimer. Structural modeling of the EGFR-L861Q mutant has also unveiled a considerable conformational rearrangement change, which is largely localized in the activation loop, while the global changes are rather moderate. We detected that a salt bridge interaction between Lys745 (P-loop) and Glu762 (*α*C-helix), present in the wild-type structure, is also maintained in the mutant model. Moreover, there is a new network of hydrogen bonds formed in L858Q, including Arg836 (catalytic loop)-Glu758 (*α*C-helix) and Ala859 (activation loop)-Val765 (*α*C-helix) interactions. The change in the interaction pattern is determined by appreciable structural changes, where the *α*C-helix and the activation loop come closer to each other (*α*C-helix moves down, the activation loop moves up, and the P-loop moves away from the *α*C-helix). Structural modeling of the L858R and L861Q mutations in EGFR points to a similar intrinsic mechanism of activation, which may imply a severe destabilization of the wild-type inactive conformation upon mutation and shifting equilibrium towards the active-like state of the enzyme, which would arguably have a detrimental effect on modulating normal kinase activity.

### 2.8. Differential Effect of Activating and Inactivating Oncogenic Mutations on the FGFR Kinases

Constitutive activation of the FGFR kinase family by a variety of mutations has been reported in several cancers [64–68]. Among frequent activating FGFR2 mutations in endometrial carcinomas are I548V, N550K, E565G, E565A, N549H, N540K, K526E, K660E, R678G, K641R, and G663E [64]. Three somatic FGFR2 endometrial mutations in the catalytic domain have an identical missense change reported in the paralogous position in FGFR3 (I538V, N540K, and K650E) in a skeletal chondrodysplasia [64]. In lung squamous cell carcinoma, the FGFR family is also one of the most frequently altered receptor tyrosine kinase families. The lung cancer activating mutations in FGFR2 include W290C, E471Q, T787K, and kinase domain mutations K660E and K660N [65]. The respective activating mutations in FGFR3 include R248C, S249C, S435C, and kinase domain mutation K717M [65]. The loss-of-function (LOF) FGFR2 kinase domain mutations identified in melanoma tumors cell lines include E636K, M640I, I642V, R759Q, E475K, D530N, A648T, and G701S [66]. Targeted genetic dependency screen is an efficient approach to identify somatic cancer alterations and was used to identify gain-of-function (GOF) mutations in lung cancer for FGFR4, MAP3K9, and PAK5 kinases [119]. Among discovered mutations were activating FGFR4 mutations P712T, H713R, S772N, and D127H, as well as a kinase-dead alteration K503M [119].

**Figure 10:**
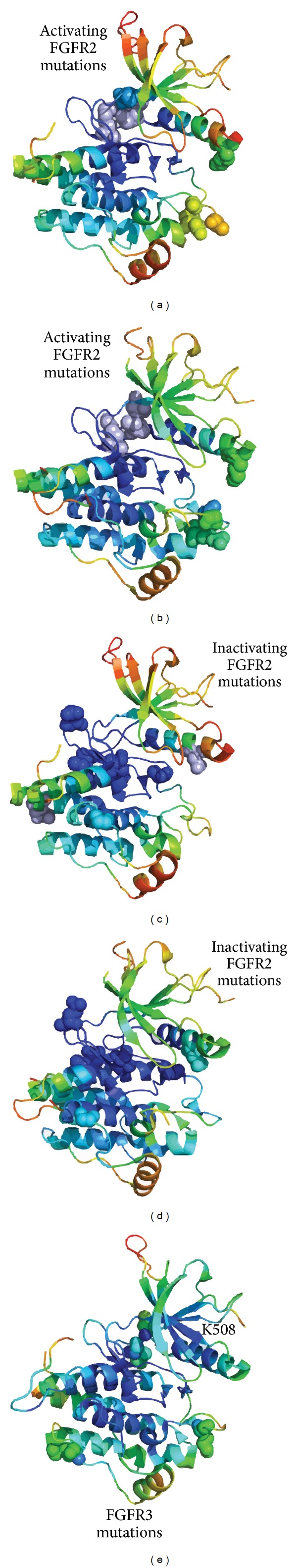
Functional dynamics and structural map of the FGFR2 and FGFR3 cancer mutations. Functional dynamics and conformational mobility map of the EGFR2 activating mutations (I548V, N550K, E565G, N549H, N540K, K526E, K660E, R678G, K641R, and G663E) projected onto the unphosphorylated FGFR2 crystal structure, pdb id 2PSQ (a) and phosphorylated active FGFR2 crystal structure, pdb id 2PVF (b). Dynamic maps of the EGFR2 inactivating mutations (E636K, M640I, I642V, R759Q, E475K, D530N, A648T, and G701S) projected onto the unphosphorylated FGFR2 crystal structure (c) and the phosphorylated active FGFR2 crystal structure (d). Dynamic map of the FGFR3 activating mutations (I538V, N540K, and K650E) and inactivating mutation (K508) projected onto the crystal structure of FGFR3 (pdb id 4k33) (e). A surface-based protein representation is employed, colored (blue-to-red) according to the protein residue motilities (from more rigid-blue regions to more flexible-red regions).

Mapping of the FGFR2/FGFR3 cancer mutations onto the crystal structures and conformational dynamics profiles demonstrated that mutations are broadly distributed in the kinase domain occupying different segments (Figure 10). Despite this diversity, we observed a subtle yet important distinction between dynamics profiles of the activating and kinase-dead mutations. The activating FGFR2 mutations simulated using the unphosphorylated (Figure 10(a)) and phosphorylated crystal structure (Figure 10(b)) are typically located either in flexible regions or at the borders between structurally stable and mobile regions. In contrast, the kinase-dead FGFR2 mutations are associated with the structurally stable and predominantly rigid segments of the kinase domain (Figures 10(c) and 10(d)). Some of the FGFR2 inactivating mutations target hydrophobic residues M640, I642, and A648 residues located near the *β*8 strand that precedes the activation loop of the kinase domain. Structural stability of these residues is determined by their interactions with the *α*E-helix in the innermost core of the C-lobe of the kinase domain. As a result even modest substitutions of these residues with the smaller hydrophobic side chains would disturb the integrity of the catalytic core of the kinase and lead to severe alterations in the kinase function and loss of activity. On the other hand, the relatively high mobility of kinase residues whose alterations lead to activating mutations can be explained by their proximity to the borders of high and low stability regions and hinge sites. Indeed, the internal mobility of these sites coupled with their prime location critical for coordinating global motions of the kinase domain could facilitate conformational transitions between inactive and active conformations. Our results suggest that the observed structural and dynamic signatures of activating mutation sites could reduce a steric barrier for the movement of the kinase N-lobe toward the C-lobe, which takes place when the kinase transits from the basal “low” activity state to the “active” state.

### 2.9. Structural and Dynamic Analysis of Cancer Mutations in BRAF: Kinase-Inactivating Mutations Stabilize the Inactive Conformation

The kinase-dead mutant forms of the Asp residue from the DFG motif have been found in various kinases where the carboxyl oxygen of this highly conserved residue plays a critical role in chelating Mg^2+^ and stabilizing ATP binding in the catalytic site [60–63]. Mutations of this Asp to residues that are not negatively charged and thus cannot chelate the Mg^2+^causing kinase inactivation. These mutations form a structurally conserved hotspot (Table 1) shared by many essential kinases including BRAF-D594V [62, 63], DAPK3-D161N [71], HCK-D378G [60, 61], and LYN-D385Y [60, 120]. The glycine residue from the DFG motif, which is adjacent to the Asp residue, is another important mutational hotspot of kinase inactivating mutations and includes BRAF-G596R, FYN-G410R, EPHA3-G766E, MAPK8-G171S, CDK11-G175S, and MAST205-G655A [60, 61]. Although most BRAF mutants display elevated kinase activity compared to the wild type, four cancer-derived mutants have reduced kinase activity: G466E, G466V, G596R, and D594V. Among the naturally occurring mutations, two that involve amino acids of the conserved DFG motif in the activation loop D594V and G596R are kinase-inactivating alterations. However, BRAF can promote MEK-ERK activation and tumor progression through several mechanisms, including the proposed mechanism of tumorigenesis mediated by kinase-dead BRAF that could cooperate with oncogenic RAS to induce CRAF activation and MEK-ERK signaling [63]. Most recently, Taylor and coworkers have put forward a mechanism of allosteric activation of functionally asymmetric RAF dimers [121] where the assembly of the catalytic and the regulatory spines provides a unifying explanation for protein kinase activation [82, 83]. Remarkably, a single mutation in the regulatory spine could assemble the active conformations bypassing the entire regulatory pathway involving cooperation with RAS, dimerization step, various phosphorylation events, and allosteric binding to regulatory partners [121].

**Figure 11:**
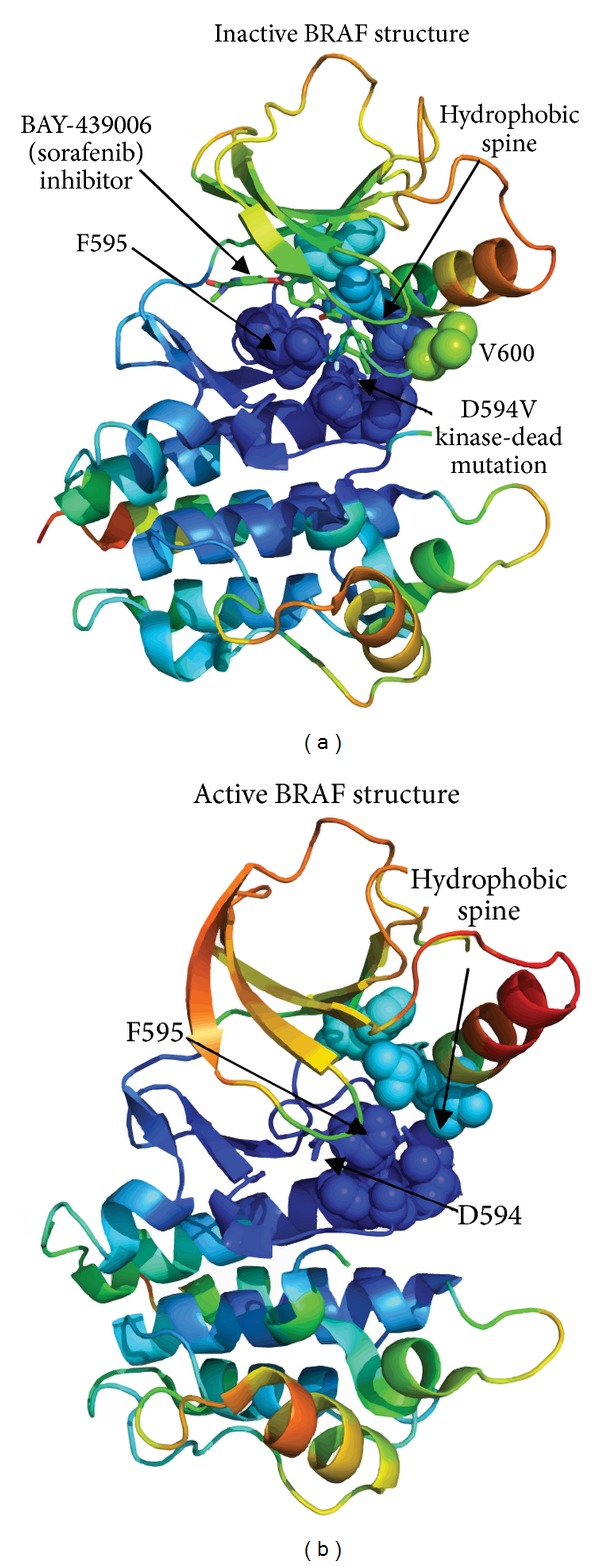
Functional dynamics and structural map of cancer mutations in the inactive and active BRAF structures. Functional dynamics and conformational mobility maps of the kinase-dead mutation D594V in the inactive BRAF crystal structure, pdb id 1uwh (a) and activating V600E mutation in the active BRAF crystal structure of BRAF kinase domain, pdb id 3c4c (b). Structural distribution of the protein kinase mobility was obtained from MD simulations and is averaged over the two lowest frequency modes. The hydrophobic spine residues (F516-L505-I513-L567-H574-I572-V504) are shown in spheres. The D594V residue (a) and D594 residue (b) are shown in sticks and pointed by arrow. The F595 residue from the DFG motif is shown in spheres and annotated. The position of the BRAF selective inhibitor sorafenib (in sticks, atom-based coloring) is shown in (a). The hydrophobic spine is partially disassembled in the inactive form (a) as F595 is flipped in DFG-out position. The spine is fully assembled in the active conformation (b).

We analyzed the structural and dynamic effects of the D594V mutation on the crystal structures of BRAF kinases in the context of a possible interplay between the site of mutation and regulatory spine. In accordance with the initial experimental conjecture [63] we suggested that the kinase-inactivating D594V mutation could mimic selective BRAF inhibitors by stabilizing the catalytically inactive BRAF conformation. This may cause the interference with the assembly of the regulatory spine and thus block the formation of the active conformation. The kinase-inactivating BRAF-D594V and activating BRAF-V600E were constructed using the crystal structures of BRAF in the inactive (pdb id 1uwh, 1uwj) [24] and active states (pdb id 3c4c) [121]. In each case, we also analyzed the dynamics preferences and stability of the hydrophobic spine residues (Figure 11). Functional dynamics analysis and structural maps of conformational mobility demonstrated that the kinase-dead D594V mutation may stabilize the inactive state of BRAF (Figure 11(a)). D594 which has a negatively charged polar side chain is thus replaced with Val that has a nonpolar side chain. As a result of this mutation, the hydrophobic side chain of the D594V residue interacts with the hydrophobic spine and together with F595 could restrict the flexibility of the activation loop and partly mimic the structural effect of sorafenib which is high affinity selective inhibitor stabilizing the inactive conformation of BRAF (Figure 11(a)). This substitution may induce stabilization of the F595 conformation which could block the ATP binding site in a similar way as sorafenib. In agreement with earlier studies [122–124], we found that D594V cooperates with F595 to stabilize the activation loop in the catalytically unproductive conformation, thus resulting in the autoinhibitory mechanism. One could also observe that this region in the inactive conformation is formed by structurally stable residues. This suggests that kinase-dead mutations may occur in the regions of higher structural stability and in the case of BRAF D594V hydrophobic modification could increase thermodynamic stability of the inactive kinase form. As a result, this effect may also increase steric barrier for conformational transitions to the active conformation, thus rendering D594V as a kinase-inactivating mutation. Concurring with our previous findings, we also observed that the activating BRAF-V600E mutation corresponds to a fairly flexible region of the catalytic domain, making this site prone to conformational changes that could facilitate transitions to the active form of BRAF (Figure 11(b)).

### 2.10. Structural and Dynamic Effects of Kinase-Inactivating Mutations: Convergent Functional Solutions by Targeting Structurally Stable Residues

**Figure 12:**
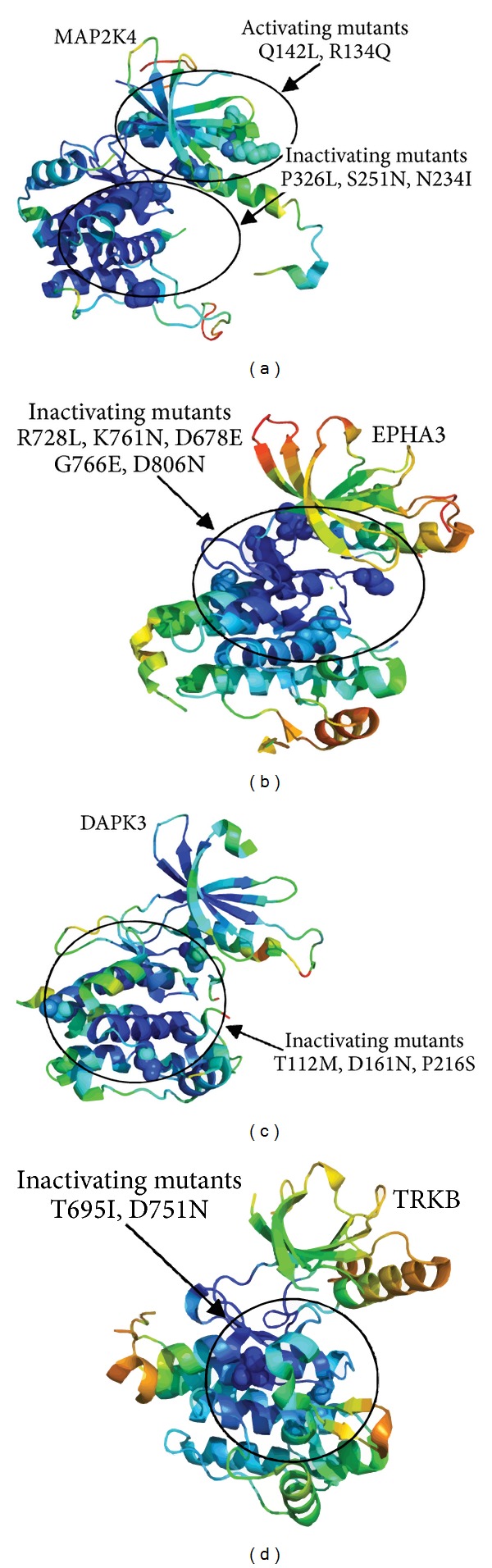
Functional dynamics and structural map of inactivating cancer mutants in different kinase genes. Functional dynamics and conformational mobility maps of the kinase mutants were computed using MD simulations of the crystal structures. (a) The dynamic profile of the MAP2K4 kinase (pdb id 3alo) with the mapped activating mutations (Q142L, R134Q) and inactivating mutations (P326L, S251N, and N234I). (b) The dynamics profile of EPHA3 (pdb id 2qoq) with the mapped inactivating mutations (R728L, K761N, D6778E, G766E, and D806N). (c) The dynamics profile of the DAPK3 kinase (pdb id 3bhy) with the mapped inactivating mutations T112M, D161N, and P216S. (d) The dynamics profile of the TRKB kinase (pdb id 4asz) with the mapped inactivating mutations T695I and D751N. A surface-based protein representation is employed, colored (blue-to-red) according to the protein residue motilities (from more rigid-blue regions to more flexible-red regions).

To provide a substantiated comparative analysis of kinase-inactivating mutations, we compared the effect of these mutations on conformational mobility in different kinases including MAP2K4 [69], EPHA3 [70], DAPK3 [71], and TRKB [72]. In case of MAP2K4 kinase, the results of activation of Q142L and R134Q were similar to those seen with the wild type, whereas the most inactive mutants R154W, P326L, S251N, and N234I produced markedly attenuated kinase activity [69] (Figure 12(a)). Consistent with previous observations, we found that kinase-inactivating mutations in MAP2K4 may assemble in structurally stable regions, while activating mutations or mutants with similar activation as in the wild-type kinase target more flexible regions (Figure 12(a)).

EPHA3 mutations are implicated in lung cancer and are distributed throughout the domains of EPHA3 (Figure 12(b)) [70]. We mapped kinase-inactivating mutations R728L, K761N, D678E, G766E, and D806N onto conformational dynamics profile of EPHA3 and monitored their effect on structural stability of the catalytic core. Similarly, we also observed that mutational sites which abolish EPHA3 activity correspond to structurally rigid regions, where drastic alterations may have a devastating effect on kinase function. Moreover, some mutations K761N, G766E, and D806N are also conserved in tyrosine kinases. Mutations of these residues may strongly affect structural stability of the catalytic core and thus kinase function (Figure 12(b)). G766 is located in an absolutely conserved DFG segment of the activation loop and is predicted to affect kinase activity. D806 is situated in the middle of kinase domain and forms two hydrogen bonds with the neighboring residues. Both G766E and D806N mutations discovered might be expected to disrupt the folding of the kinase domain.

DAPK3 is a relatively novel cancer-associated kinase with functional mutations. Evaluation of nonsynonymous DAPK3 point mutations in various tumors (T112M, D161N, and P216S) reveals that all three mutations decrease or abolish kinase activity [71]. These DAPK3 mutations identified in cancer patients can significantly suppress the activity of the kinase. According to our analysis, these mutations have a combined pattern of rigidity/mobility and cannot be unambiguously assigned to exclusively rigid segments of the catalytic domain. The broad distribution of these mutations and their position at the C-terminal lobe segments implicated in protein binding could indicate that loss of activity in DAPK3 is unlikely associated with the effect on kinase fold but rather on binding with regulatory partners. TRK kinases include the three highly homologous proteins TRKA, TRKB, and TRKC that are strongly associated with central and peripheral nervous system processes. Recently, colon cancer-derived mutants, TRKB-T695I and TRKB-D751N, demonstrated a significantly reduced activity compared to that of wild-type TRKB. Consistently, upon stimulation these mutants were impaired in activating TRKB and its downstream effectors AKT and ERK [72]. In simulations, we used recently revealed first crystal structures of the TRKB kinase domain in apo form and complexes with high affinity inhibitors [125]. Our analysis suggested that structural map of kinase-inactivating mutations is associated with structurally rigid residues both inside the catalytic core and belong to the most structurally stable helices (T695I and D751N), whereas D751N is also proximal and to the protein-binding region in the C-terminal lobe (Figure 12(d)). As a result, this may rationalize the fact that these mutants were essentially irresponsive to activation stimulus by TRKB ligand BDNF [72].

## 3. Discussion

The results of our study suggest that that somatic mutations occurring at structurally conserved positions within the protein kinase catalytic domain may be statistically enriched in cancers and form mutational hotspots that promote the tumorigenic activity of multiple protein kinases. The majority of activating cancer mutations cause only moderate structural changes, but may produce considerable local rearrangements in the conformation of the activation loop. The appreciable structural changes in the activation loop for activating mutations are exemplified by conformational transitions to the active-like form of the protein kinase. The discovered differences in structure and energetics between the wild-type kinases and cancer mutants point to a common mechanism of constitutive activation, which may be determined by a combined effect of the partial destabilization of the inactive state and a concomitant stabilization of the active-like form of the enzyme. Since these structures are known natural protein conformations existing in a dynamic equilibrium, we suggest that cancer mutations may trigger conformational transitions which mimic the activation process of the normal kinase. Our results also demonstrated that many kinase-inactivating mutations across different kinase genes are often located in structurally rigid sites where mutations could either stabilize the autoinhibitory inactive state and prevent activation or severely reduce kinase activity through detrimental changes in the kinase fold. These findings are in excellent agreement with the recent analysis of structural effects of cancer-associated missense mutations in oncogenes [126]. These studies determined that oncogenic mutations are less often destabilizing and functional sites are more often subject to cancer mutations. Interestingly, it was similarly concluded that mutations located in the protein core are often destabilizing and may therefore result in loss-of-function. Our results corroborate with these studies and collectively could offer a plausible model for explaining the mechanism of inactivating mutations in oncogenic proteins beyond protein kinases. While the structural diversity of wild-type protein kinases has been illuminated in recent years given the rapidly increasing body of crystal structures, structural knowledge of functionally important kinase mutants is limited and presents an important challenge for “disease-oriented” structural studies of protein families. It is evident that the accuracy of computational structure predictions for kinase cancer mutants and the atomic details of activation mechanisms can be fully understood only when the respective high resolution crystal structures become available. However, considering current experimental challenges in dissecting the molecular basis of cancer causing mutations, the presented evolutionary and structural analyses of the mutational hotspots in protein kinases provide useful insights into the mechanistic basis of activation which agree with available experimental data.

Thus, computational predictions from this study can inform and facilitate experiments exploring the molecular pathology of tumorigenesis and implications in rational drug design of specific cancer therapies. In particular, characterization of the conformational landscape for the wild-type protein kinases and cancer mutants can provide access to unique inactive conformations, otherwise hidden in biochemical and structural experiments typically biased towards the active state of the enzyme. If such alternative states can be identified and targeted effectively, structural models of cancer mutants may be employed for identifying new indications, as well as reengineering and optimizing the clinical effects of existing drugs. The emerging understanding of evolutionary and molecular signatures, associated with cancer causing mutations in protein kinases, may be also useful for design of personalized agents which target a spectrum of specific mutational changes occurring in cancer.

## 4. Materials and Methods

### 4.1. Sequence Analysis

Disease causing, somatic, and common SNPs were obtained and mapped to kinase sequences as described in [46, 48]. Disease causing variants represent a subset of nscSNPs which are found to cause an inherited disease, while somatic mutations are found in tumors, and common SNPs are referred to as the ones which have not been implicated in disease and are somewhat common in the population. In total, we examined 355 common SNPs, 428 inherited disease causing SNPs, and 541 cancer associated SNPs. The SNPs were chosen to be composed of a nonredundant set of SNPs, such that no site within a particular kinase was counted more than once. Known somatic driver mutations were obtained by searching OMIM [117]. Somatic and germline mutations from cancer cell lines were obtained from the kinome resequencing study [60]. The catalogue of observed somatic mutations was obtained from the cosmic database [118]. Disease causing and common SNPs were obtained and mapped to kinase sequences as described in [49]. A nonredundant set of SNPs was generated so that no site within a particular kinase was counted more than once. In total, 428 disease causing SNPs and 330 common SNPs were compiled for the analyses.

Kinase sequences were aligned to characteristic catalytic site motifs. Regions are denoted based on the definitions provided by Hanks and Hunter [1], where a denotes the intervening region between subdomains. Note that subdomain X is split into two halves, X(i) and X(ii). The expected probability (*E*(*p*)) of an SNP occurring in a region was calculated separately for common and disease SNPs as follows. The average length of each region was calculated as the weighted average of the region length in each kinase considered, where weights correspond to the total number of SNPs occurring within each kinase. This weighting helps avoid biases that might arise as a result of some kinases simply harboring more SNPs than others. The probability of an SNP occurring within a particular region purely by chance was computed as its weighted average length over the sum of every region's weighted average length. The probability (*P* value) of the observed total number (*x*) of SNPs occurring within each region, where *n* is the total number of SNPs considered, was calculated using the general binomial distribution as follows:

 If *x*/*n* < *E*(*p*):
  (1) $$\begin{array}{c} P\;\;\text{value}\left(x\right)=\left(\overset{x}{\underset{x=0}{\sum }}\left(\begin{array}{c} n\\ x \end{array}\right)\cdot E{\left(p\right)}^{x}{\left(1-E\left(p\right)\right)}^{n-x}\right)\cdot 2. \end{array}$$
 If *x*/*n* > *E*(*p*):
  (2) $$\begin{array}{c} P\;\;\text{value}\left(x\right)=\left(\overset{n}{\underset{x=x}{\sum }}\left(\begin{array}{c} n\\ x \end{array}\right)\cdot E{\left(p\right)}^{x}{\left(1-E(p)\right)}^{n-x}\right)\cdot 2. \end{array}$$

Comparisons of the average length per region in the common and disease SNPs sets, as well as the comparison of the number of SNPs per region, and the number occurring within subdomains versus intervening regions were calculated using the normal distribution approximation to the binomial distribution. Multiple alignments were generated by using a motif model. Sites with multiple disease SNPs were considered for further structural analysis. To estimate whether disease SNPs are position-specific or distributed randomly throughout the catalytic domain, in addition to a pairwise correlation, we ran 10,000 Monte Carlo simulations involving random assignment of disease SNPs. The SNP distribution resulting from this simulation study compared to the observed distribution was that zero SNPs occurred at an average of 19.52 ± 0.03 positions in the simulation versus 46 observed positions; 1 SNP at 67.58 ± 0.06 positions versus 65 observed positions; 2 SNPs at 76.95 ± 0.07 positions versus 47 observed positions; 3 SNPs at 35.04 ± 0.04 positions versus 18 observed positions, 4 SNPs at 7.20 ± 0.03 positions versus 21 observed positions, 5 SNPs at 0.69 ± 0.01 positions versus 3 observed positions, 6 SNPs at 0.03 ± 0.002 positions versus 3 observed positions, 7 SNPs at 0.0002 ± 0.0001 positions versus 3 observed positions, and 8 SNPs at 0.0 ± 0.0 positions versus 1 observed position. The average length of each subdomain was calculated as the weighted average of the region length in each kinase considered, where weights correspond to the total number of SNPs occurring within each kinase. The probability of an SNP occurring within a particular region purely by chance was computed as its weighted average length is divided by the sum of every region's weighted average length. The probability (*P* value) of the observed total number of SNPs occurring within each region was then calculated using the general binomial distribution. Cancer mutant predictions and analysis were performed as described in [47]. Briefly, a support vector machine (SVM) was trained upon common SNPs (presumed neutral) and congenital disease causing SNPs characterized by a variety of sequence, structural, and phylogenetic parameters [47]. The threshold taken for calling an SNP a driver is 0.49 for catalytic domain mutations and 0.53 for all other mutations as described in [47].

### 4.2. Structural Predictions of Kinase Cancer Mutants

Structure predictions of kinase cancer mutants were done using MODELLER [127] with optimization of side chains by the SCRWL3 program [128]. The models were then refined using 5000 steps of minimization and 2 ns MD simulations with the NAMD 2.6 software package [129]. Initial models were built in MODELLER with a flexible sphere of 5 (Angstrom, “Å”) around mutated residue. In the final protocol, we gradually increased the radius of this sphere in 5 Å steps until the radius reached the 25 Å values (i.e., all residues falling within this range were treated as flexible). A hybrid protocol involving a conjugate gradient (CG) minimization, followed by MD simulations with simulated annealing refinement, was repeated 20 times to generate 100 initial models for each cancer mutant in this study. In the optimization stage, we initially used a conjugate gradient (CG) minimization to remove unfavorable contacts and to optimize geometry. MD simulations were then run at increasing temperature values from 150 K to 1500 K, followed by simulated annealing and sampling at temperatures of 1500 K, 1000 K, 800 K, 600 K, 500 K, 400 K, 320 K, and 300 K, respectively. The models were generated using 20 iterations of the MD/SA procedure and the predicted structural model was chosen out of the 100 models as scored by the MODELLER default scoring function.

### 4.3. MD Simulations

5 ns MD equilibrium simulations were carried out on the crystal structures and refined structural models of cancer mutants using NAMD 2.6 [129] with the CHARMM27 force field [130, 131] and the explicit TIP3P water model as implemented in NAMD 2.6 [132]. The VMD program was used for the preparation and analysis of simulations [133, 134]. The employed MD protocol was described in full details in our earlier studies [135–137]. In brief, structures were solvated in a water box with the buffering distance of 10 Å. The system was subjected to initial minimization for 20,000 steps (40 ps) keeping protein backbone fixed which was followed by 20,000 steps (40 ps) of minimization without any constraints. Equilibration was done in steps by gradually increasing the system temperature in steps of 20 K starting from 10 K until 310 K and at each step 15000 steps (30 ps) equilibration was run keeping a restraint of 10 Kcal mol^−1^ Å^−2^ on protein alpha carbons (C_*α*_). Thereafter the system was equilibrated for 150,000 steps (300 ps) at 310 K (NVT) and then for further 150,000 steps (300 ps) at 310 K using Langevin piston (NPT) to achieve uniform pressure. Finally the restrains were removed and the system was equilibrated for 500,000 steps (1 ns) to prepare the system for simulation. An NPT simulation was run on the equilibrated structure for 20 ns keeping the temp at 310 K and pressure at 1 bar using Langevin piston coupling algorithm. Nonbonded van der Waals interactions were treated by using a switching function at 10 Å and reaching zero at a distance of 12 Å.

### 4.4. Principal Component Analysis

Protein flexibility was analyzed by combining the results of MD simulations with the principal component analysis of conformational ensembles [138, 139]. The covariance matrix between residues (represented by the C_*α*_ atoms) *i* and *j* was calculated for each of the 20 ns MD simulation trajectories: snapshots from trajectories were taken every 200 ps, overall translation and rotation were removed, and only C_*α*_ was kept for analysis.

To obtain collective motion coordinates that represent the overall dynamics of each trajectory, PCA was performed, in which the covariance matrix $C_{ij}=\langle \left({\vec{r}}_{i}-{\vec{r}}_{i,\text{ave}}\right)\cdot \left({\vec{r}}_{j}-{\vec{r}}_{j,\text{ave}}\right)\rangle$ was diagonalized to yield a set of eigenvectors and eigenvalues. The correlation matrix represents the correlation between the motion of atom *i* and of atom *j*, obtained from the normalization of the covariance matrix:

(3) $$\begin{array}{c} {\text{Corr}}_{ij}=\frac{\langle \left({\vec{r}}_{i}-{\vec{r}}_{i,\text{ave}}\right)\cdot \left({\vec{r}}_{j}-{\vec{r}}_{j,\text{ave}}\right)\rangle }{\sqrt{{\langle {\vec{r}}_{i}-{\vec{r}}_{i,\text{ave}}\rangle }^{2}{\langle {\vec{r}}_{j}-{\vec{r}}_{j,\text{ave}}\rangle }^{2}}}. \end{array}$$

The eigenvectors represent the directions in the multidimensional space that correspond to independent modes of atomic motion, while the eigenvalues represent their corresponding amplitudes [138, 139]. The principal components of protein motions are analyzed by projecting MD trajectories onto directions corresponding to the largest eigenvectors. The correlation value is the normalized covariance matrix, ranging from −1 to 1. The calculations were performed using the CARMA package [140] and PCA_NEST web-based service [141].

## Figures and Tables

**Table 1:** Structurally conserved cancer mutation hotspots in protein kinase genes.

Reference (EGFR)	Segment	Number of mutations	Number of drivers	Mutations	Activation status
L718	P-loop	4	4	ABL L248V	Activating
				BRAF I462S	
				EGFR L718P	
				JAK3 L527P	

G721	P-loop	5	4	EGFRG721	Activating
				BRAF G464A/E/R/V	
				caMLCK G601E	
				MLK2 G107E	
				NDR2 G99A	
				AurC G52E	

T790	Hinge	6	5	EGFR T790M	Activating
				ABL T315I/N	
				FGFR4 V550M	
				KIT T670E	
				PDGFRa T674I	
				NEK11 T108M	

G796	Hinge	5	5	EGFR G796S	Activating
				ABL G321E	
				ErbB2 G804S	
				IRR G1065E	
				LKB1 G135R	

D855	Activation	5	5	BRAF D594E	Inactivating
				DAPK3 D161N	
				HCK D378G	
				LKB1 D194V	
				LYN D385Y	

G857	Activation	6	5	BRAF G596R	Inactivating
				EPHA3 G766E	
				FYN G410R	
				JNK1 G171S	
				MAST2 G655A	
				CDK11 G175S	

L858	Activation	3	3	EGFR L858R	Activating
				BRAF L596V	
				ErbB2 L866R	

L861	Activation	8	8	EGFR L861Q/R	Activating
				ABL L387M	
				BRAFV600D/E/G/K/L/M	
				ErbB2 L869Q	
				FLT3 D835E/F/H/N/V/Y	
				KIT D816E/F/V/H/I/N/V	
				MET D1246H/N/V	
				PDGFRa D842I/V/Y	

**Table 2:** Summary of modeled somatic kinase mutations.

Kinase	Mutations	Activation status
ABL	L248V, T315I/L/N	Activating
	G321E, Y353H, L387M	Activating

EGFR	L718/P	Activating
	G719C/A/R/D/V/S	Activating
	G721D	Activating
	L747S/P/F	Activating
	V765M, V769L, T790M	Activating
	G796S, L833V, T854A	Activating
	D855	Inactivating
	L858R, L861Q/R	Activating

BRAF	G466E/V, D594E/V, G596R	Inactivating
	L597V/S/Q/R/L	Activating
	A598V, T599I/A, V600E/K/R/M	Activating

FGFR2	I548V, N550K, E565G, E565A	Activating
	N549H, N540K, K526E,	Activating
	K660E, R678G, K641R, G663E	Activating
	E636K, M640I, I642V, R759Q, E475K	Inactivating
	D530N, A648T, G701S	Inactivating

FGFR3	I538V, N540K, K650E	Activating
	K508	Inactivating

FGFR4	P712T, H713R, S772N, D127H	Activating
	K503M	Inactivating

MAP2K4	Q142L, R134Q	Activating
	R154W, P326L, S251N, N234I	Inactivating

DAPK3	T112M, D161N, P216S	Inactivating

HCK	D378G	Inactivating

LYN	D385G	Inactivating

LKB1	D194V	Inactivating

FYN	G410R	Inactivating

EPHA3	R728L, K761N, D678E, G766E, D806N	Inactivating

MAPK8	G171S	Inactivating

CDK11	G175S	Inactivating

MAST205	G655A	Inactivating

TRKB	T695I, D751N	Inactivating

ERBB2	I767M, D769H/Y, V773AV777L,	Activating
	T798M, G804S, Y835F, V842I,	Activating
	T862A, L866R, L869Q, R896C	Activating
	K753M	Inactivating

ERBB3	V714M, Q809R, V855A, S846I, E298C	Activating

ERBB4	V696I, A748S, E785K, R757Q, P829Q, T901M	No effect on kinase activity
	K726R, D818N, D836Q	Inactivating
